# Home visits and the perception of health status among residents in Peru, 2024

**DOI:** 10.15649/cuidarte.4238

**Published:** 2025-08-14

**Authors:** Janett Virginia Chavez Sosa

**Affiliations:** 1 Universidad Peruana Unión, Lima, Peru. E-mail: viki16@upeu.edu.pe Universidad Peruana Unión Lima Peru viki16@upeu.edu.pe

**Keywords:** Quality of Health Care, Home Visit, Perception, Health Status, Calidad de la Atención de Salud, Visita Domiciliaria, Percepción, Estado de Salud, Qualidade da Assistência à Saúde, Visita Domiciliar, Percepção, Nível de Saúde

## Abstract

**Introduction:**

Nursing home visits play a key role in healthcare by providing personalized care at home. However, their impact on the population's perception of health status remains not yet fully understood.

**Objective:**

To determine the relationship between the quality of nursing home visits and the perception of health status in the Peruvian population.

**Materials and Methods:**

An analytical, cross-sectional study was conducted with 482 people. The "Home Visits" and "SF-36" questionnaires were used to collect data. The analysis was carried out using binary logistic regression.

**Results:**

A total of 91.49% of participants reported an adequate perception of their health status, and 90.88% rated the quality of home visits as good. Bivariate analysis revealed significant differences according to age (p=0.005), having children (p=0.001), and the quality of home visits (p=0.001). In the multivariate analysis, having children (OR: 4.96; 95% CI: 1.38-17.74) and receiving good-quality home visits (OR: 20.79; 95% CI: 9.12-47.42) were associated with a more favorable perception of health status.

**Discussion:**

These findings suggest that higher-quality home visits are associated with a more favorable perception of health status, aligning with previous studies on the importance of home care.

**Conclusion:**

Strengthening the quality of home visits could improve the population's perception of health.

## Resumen

**Introducción:**

Las visitas domiciliarias de enfermería desempeñan un papel clave en el cuidado de la salud al proporcionar atención personalizada en el hogar. Sin embargo, su impacto en la percepción del estado de salud de la población aún no está completamente esclarecido.

**Objetivo:**

Determinar la relación entre la calidad de las visitas domiciliarias de enfermería y la percepción del estado de salud en población peruana.

**Materiales y métodos:**

Se realizó un estudio analítico y de corte transversal con una muestra de 482 personas. Se utilizaron los cuestionarios “Visitas Domiciliarias” y el “SF-36” para la recolección de datos. El análisis se llevó a cabo mediante regresión logística binaria.

**Resultados:**

El 91,49% de los participantes reportó una buena percepción de su estado de salud y el 90,88% calificó la calidad de la visita domiciliaria como buena. El análisis bivariado reveló diferencias significativas según la edad (p=0,005), tener hijos (p=0,001) y la calidad de las visitas domiciliarias (p=0,001). En el análisis multivariado, tener hijos (OR: 4,96; IC 95%: 1,38-17,74) y una buena calidad de las visitas domiciliarias (OR: 20,79; IC 95%: 9,12-47,42) se asociaron con una mejor percepción del estado de salud.

**Discusión:**

Los hallazgos sugieren que una mayor calidad en las visitas domiciliarias se relaciona con una mejor percepción del estado de salud, lo que coincide con estudios previos sobre la importancia del cuidado domiciliario.

**Conclusiones:**

Fortalecer la calidad de las visitas domiciliarias podría mejorar la percepción de salud de la población.

## Resumo

**Introdução:**

As visitas domiciliares de enfermagem desempenham um papel fundamental nos cuidados de saúde, pois proporcionam cuidados personalizados em casa. Entretanto, seu impacto na percepção da população sobre o estado de saúde ainda não é totalmente compreendido.

**Objetivo:**

Determinar a relação entre a qualidade das visitas domiciliares de idosos e a percepção do estado de saúde na população peruana.

**Materiais e métodos:**

Foi realizado um estudo analítico e transversal com uma amostra de 482 pessoas. Para coleta de dados foram utilizados os questionários “Visitas Domiciliares” e “SF-36”. A análise foi realizada por meio de regressão logística binária.

**Resultados:**

91,49% dos participantes relataram uma boa percepção do seu estado de saúde e 90,88% classificaram a qualidade da visita domiciliar como boa. A análise bivariada revelou diferenças significativas conforme a idade (p=0,005), a presença de filhos (p=0,001) e a qualidade das visitas domiciliares (p=0,001). Na análise multivariada, ter filhos (OR: 4,96; IC 95%: 1,38-17,74) e boa qualidade das visitas domiciliares (OR: 20,79; IC 95%: 9,12-47,42) foram associados à melhor percepção do estado de saúde.

**Discussão:**

Os resultados sugerem que visitas domiciliares de maior qualidade estão associadas a uma melhor percepção do estado de saúde, o que é consistente com estudos anteriores sobre a importância do atendimento domiciliar.

**Conclusões:**

O fortalecimento da qualidade das visitas domiciliares pode melhorar a percepção de saúde da população.

## Introduction

Approximately 4.5 billion people worldwide, or half of the global population, lack full access to essential health services^1^. The majority of interventions (90%) needed to achieve universal health coverage can be implemented using a primary health care approach. This approach is estimated to contribute to 75% of the progress envisaged in the Sustainable Development Goals^2^. Therefore, the World Health Organization, through the Astana Declaration, advocates for health education and the fulfillment of individuals and communities' expectations for reliable health information^3^.

In this context, home visits represent a key intervention within the primary health care approach, as they allow health professionals to provide services directly to patients in their homes^4^. This is especially crucial for individuals who face geographic, economic, or social barriers to accessing health facilities^5^. Studies in various countries have shown that home visits made by health professionals can improve adherence to treatments^6^, chronic diseases management^7^, and patients' quality of life^8^. However, their implementation also implies increased health expenditure, posing sustainability challenges. Therefore, it is essential to rethink the health system to ensure it responds adequately to society's new health needs and promotes a sustainable public healthcare system for the future. Given this situation, the Federation of Associations of Community Nursing and Primary Care (FAECAP) has proposed that community nurses are the best-positioned professionals to carry out these initiatives cost-effectively^9^. 

Beyond their clinical benefits, home visits can influence individuals' perception of their health status. This perception is a multidimensional aspect that involves a subjective evaluation of physical, mental, and social well-being. Factors such as chronic diseases, access to healthcare services, family support, and professional interventions can influence how individuals perceive their health status^10^,^11^. Home visits can significantly improve this perception by providing health education, support, early problem detection, and continuity of care^12^,^13^.

In Latin America, the implementation of home visiting programs varies significantly among countries. In Brazil, studies have shown that home care enables a comprehensive approach and ongoing care, underscoring the importance of personalized, high-quality care linked to a more favorable perception of health status^14^. In Peru, however, home visits are often conducted by technical staff or health promoters who may lack the right profile for a comprehensive intervention. As a result, these visits focus only on the delivery of supplies, which can affect the quality of care^15^.

Peruvian regulations recognize that home visits allow one to understand families within their natural environment and directly evaluate their living conditions and interactions, which are crucial elements for designing appropriate interventions. Through observation and support, home visits seek to empower caregivers and foster relationships of trust and respect between healthcare professionals and families. In addition, the quality of care provided during these visits can influence patients' perceptions of their health status. Well-structured interventions that include health education, continuous follow-up, and adequate communication can strengthen patients' confidence in their health and the health services available^16^.

Therefore, the study aimed to determine the relationship between the quality of nursing home visits and perceived health status among the Peruvian population.

## Materials and Methods

**Type of Study**

Analytical, cross-sectional study.

**Population and sample**

The study was conducted in the Virgen del Carmen La Era Human Settlement, located in the Ñaña area of the Lurigancho-Chosica district, in 2024. It lies on the right bank of the Rímac River and is part of an expanding urban area characterized by accelerated population growth and limited access to health services. According to the latest available census, this area has an estimated population of 16,360, mainly composed of middle- and low-income families that are highly dependent on primary health care. 

To determine the sample size, a 5% margin of error and a 95% confidence level were considered, resulting in a minimum required sample of 376 participants. During the data collection process, a high response rate was achieved, obtaining a final sample of 482 inhabitants who met the following inclusion criteria: Peruvian residents aged 18 years or older who had received at least one nursing home visit within the past six months and had signed the informed consent form. Likewise, foreign residents, individuals who had not received any nursing home visits, those not residing in the study area, and those who declined to participate in the study were excluded from the analysis. 

**Data collection instruments**

To evaluate the quality of nursing home visits, the "Quality of Nursing Home Care" questionnaire, created in Peru by Guerrero^17^ in 2017, was used. This instrument has a Cronbach's alpha reliability coefficient of 0.839 and consists of 27 items distributed in three dimensions: Interpersonal (items 1-13), Technical-Scientific (items 14-20), and Organizational (items 21-27). Responses were recorded on a Likert-type scale ranging from "Never" (1) to "Always" (4). A total score greater than 40 indicated good quality of home visits, while a score of 39 or lower demonstrated poor quality. On the other hand, perceived health status was measured using the SF-36 questionnaire^18^, consisting of 36 items distributed across eight dimensions: Physical functioning (10 items), physical role (4 items), bodily pain (2 items), general health perceptions (5 items), vitality (4 items), social functioning (2 items), emotional role (3 items), and mental health (5 items). Responses were recorded on a 5-point Likert-type scale. The final score varies between 0 and 100 points, with higher scores indicating an adequate perception of health status.

**Procedure**

Data collection was conducted between March and June 2024. The surveys were administered during home visits, each lasting 15 to 20 minutes. Interviewers received prior training to ensure consistency in questionnaire administration and collecting information. A standardized protocol was followed to approach participants, explain the study's objectives, and obtain informed consent. Privacy was ensured during each interview, and confidentiality of the collected data was maintained.

Data processing was performed using SPSS version 24 software. Previously, data cleaning was carried out to ensure the integrity and accuracy of the collected information. Descriptive analyses were performed using simple frequency tables. For bivariate analysis, cross tabulations, and the chi-square test were used to evaluate relationships between variables, as most were categorical and did not meet the assumption of normality based on the Kolmogorov-Smirnov test. Finally, binary logistic regression was applied, as the dependent variable, perception of health status, was dichotomized into "adequate" (1) and "inadequate" (0). Odds ratios (OR) were calculated along with their corresponding 95% confidence intervals (95% CI). The data collected is available for free access and consultation in Mendeley Data^19^.

**Ethical considerations**

The study was approved by the Ethics Committee of Universidad Peruana Unión (No 2022-CE-FCS-UPeU-019). The surveys were administered after the participants signed the informed consent form, guaranteeing the confidentiality and anonymity of the information collected.

## Results

A total of 482 residents were surveyed, comprising 63.98% men and 36.02% women. The majority of participants were adults, representing 85.52% of the sample. Regarding the place of origin, 66.67% came from the Coast, 21.35% from the Mountains, and 12.08% from the Jungle. Regarding marital status, 70.72% were married or cohabiting, while 29.28% were single, divorced, or widowed. In addition, 87.56% of respondents had children (Table 1). 

Regarding health status, 91.49% of the participants considered it good, and 8.51% perceived it as poor. Concerning the quality of home visits, 90.88% of respondents rated the care received as good and 9.12% rated it as poor. When evaluating the specific dimensions of home visits, 90.45% of participants positively assessed the interpersonal dimension, 91.29% the technical-scientific dimension, and 96.27% the organizational dimension (Table 1).

**Table 1:** Descriptive analysis of the study variables

Variables	n=482 % (n)
Sex	
Female	36.02 (174)
Male	63.98 (308)
Age	
Young (18-29 years)	14.48 (70)
Adult (30-59 years)	85.52 (412)
Place of origin	
Coast	66.67 (321)
Mountain	21.35 (103)
Jungle	12.08 (58)
Marital status	
Single/divorced/widowed	29.28 (141)
Married/Cohabitant	70.72 (341)
Do you have children?	
Yes	87.56 (422)
No	12.44 (60)
Perception of health status	
Adequate	91.49 (441)
Inadequate	8.51 (41)
Quality of home visits	
Good	90.88 (438)
Poor	9.12 (44)
Interpersonal dimension	
Good	90.45 (436)
Poor	9.55 (46)
Technical/scientific dimension	
Good	91.29 (440)
Poor	8.71 (42)
Organizational dimension	
Good	96.27 (464)
Poor	3.73 (18)

Significant differences were found in the perception of health status based on age, number of children, and the quality of home visits. Regarding age, young people represented 13.21% of the sample, and within this group, 29.27% reported an inadequate perception of health status, compared to 70.73% among adults. This difference was statistically significant (p = 0.005). Regarding the number of children, 39.02% of participants without children reported an inadequate perception of health status, while only 10.00% of those with children did so. The difference was highly significant (p = 0.001), suggesting that having children may be associated with a more favorable perception of health status, regardless of the quality of home visits. Regarding the quality of home visits, 53.66% of participants who considered care to be poor reported an inadequate perception of their health status, compared to 5.00% among those who rated care as good. This difference was also significant (p = 0.001) (Table 2). 

**Table 2:** Existing association between anxiety and depression in family caregivers of critically ill patients

Variables	Perception of health		p-value
	Adequate %(n)	Inadequate %(n)	
Sex			0.277
Female	35.40 (156)	43.90 (18)	
Male	64.60 (285)	56.10 (23)	
Age			0.005
Young	13.21 (58)	29.27 (12)	
Adult	86.79 (441)	70.73 (29)	
Place of origin			0.551
Coast	66.00 (291)	73.17 (30)	
Mountain	21.51 (95)	19.51 (8)	
Jungle	12.49 (55)	7.32 (3)	
Marital status			0.072
Single/divorced/widowed	28.07 (124)	41.46 (17)	
Married/Cohabitant	71.93 (341)	58.54 (24)	
Number of children			0.001
Yes	90.00 (397)	60.98 (25)	
No	10.00 (44)	39.02 (16)	
Quality of home visits			0.001
Good	95.00 (419)	46.34 (19)	
Poor	5.00 (22)	53.66 (22)	
Interpersonal dimension			0.001
Good	95.00 (419)	41.46 (17)	
Poor	5.00 (22)	58.54 (24)	
Technical/scientific dimension			0.001
Good	95.22 (420)	48.78 (20)	
Poor	4.78 (21)	51.22 (21)	
Organizational dimension			0.001
Good	98.40 (434)	73.17 (30)	
Poor	1.60 (7)	26.83 (11)	

p-value: Pearson's Chi-Square test

The multivariate analysis showed that having children increased 4.96 times (95% CI: 1.38-17.74) the probability of having a positive perception of health status compared to residents without children. Likewise, receiving high-quality home visits increased the likelihood of a more favorable perception of health status 20.79 times (95% CI: 9.12-47.42). However, no significant differences were found between perceived health status and sex (p = 0.221) or age (p = 0.932) (Table 3). These findings suggest that, despite the differences observed in the bivariate analysis, these variables do not significantly impact the perception of health status when other factors are controlled. 

**Table 3:** Multivariate analysis according to the perception of health status

Variables	OR	95% CI		p-value
		LL	UL	
Sex				
Female	1	(Reference)		0.221
Male	1.62	0.74	1.62	
Age				
Young	1	(Reference)		0.932
Adult	0.95	0.32	2.77	
With children				
Yes	1	(Reference)		0.014
No	4.96	1.38	17.74	
Quality of home visits				
Good	1	(Reference)		0.001
Poor	20.79	9.12	47.42	

Binary logistic regression was used. OR: odds ratio, LL: lower limit, UL: upper limit.

## Discussion

The results of this study offer a comprehensive view of how different variables influence participants' perceptions of their health status, especially highlighting the quality of home visits and the influence of the number of children on health status perception.

The study revealed that 90.88% of the participants perceived that the quality of home visits provided by nurses was good. This finding is consistent with previous studies highlighting the crucial role of home care in community health. A systematic research study indicated that home visits conducted by professional nurses are associated with reduced hospitalizations and emergency visits, along with improvements in overall health and patient satisfaction^20^. Despite these benefits, a study in Volta, Ghana, identified challenges in implementing home visits, such as the lack of training and the need to expand services, highlighting the importance of improving the quality and scope of home care^21^.

Our results also showed that a positive perception of home visits was associated with a better health status assessment. This finding reinforces the need to continue improving the quality of such services. In Madrid, a nurse-led home intervention improved the quality of life for caregivers of patients with chronic diseases, a result that is consistent with our results^22^. Similarly, a study in Iran showed that a three-month program of home visits significantly improved the quality of life and adherence to treatment in hemodialysis patients^23^. However, a study in the United Kingdom observed improvements in maternal sensitivity and infant cooperation thanks to home visits, although it found no significant differences in other measures. These results suggest that while home visits may offer specific benefits, prolonged follow-up is required to assess their full impact^24^. Collectively, these studies support the idea that high-quality home visits can positively influence the perception of health status and overall satisfaction.

Another relevant finding of our study is that participants with children reported a more adequate health perception than those without children. This phenomenon may be related to a greater motivation to stay healthy for the benefit of their children. Previous research has shown that the role of parents can provide individuals with a sense of purpose and well-being, contributing to greater life satisfaction and a positive perception of health status^25^. Children may also serve as sources of emotional and social support, strengthening the positive perception of physical and mental well-being.

While the results suggest that having children could be associated with a more positive perception of health status, this relationship is not necessarily determined by the quality of home visits. The wide confidence interval in the variables "having children" and "quality of home visits" indicates high variability in the effect estimates, suggesting the possible influence of confounding factors not contemplated in this study. Aspects such as family support and caregiving could be essential in shaping these perceptions. Therefore, future studies should consider stratifying the population based on parental status to minimize bias and obtain more accurate estimates.

## Conclusions

This study concludes that the quality of nursing home visits and the presence of children in the household are significantly associated with a more positive perception of health status among the Peruvian population. These findings underscore the importance of strengthening home care to improve community perceptions of well-being.

Likewise, key aspects to consider were identified, such as the training of healthcare personnel and the provision of adequate resources, which are essential to optimize the impact of these visits. Future studies should further explore the factors that may influence these associations and consider stratifying the study population to avoid possible bias in interpreting the results.
